# Structured Light Music for Stress Adaptation: The BDNF/TrkB Signaling Axis Mediating Neuroplasticity and HPA Axis Regulation

**DOI:** 10.3390/brainsci16090959

**Published:** 2026-09-10

**Authors:** Jingqi Le, Xuelan Shu, Pingping Jia, Tao Le

**Affiliations:** 1 School of General Education and International Studie, Chongqing Polytechnic University of Electronic Technology, Chongqing 401331, China; 2 College of Life Sciences, Chongqing Normal University, Chongqing 401331, China

**Keywords:** structured light music, auditory enrichment, BDNF/TrkB signaling axis, HPA axis regulation, neuroplastic remodeling, stress adaptation

## Abstract

**Highlights:**

**What are the main findings?**

- Structured light music with defined acoustic parameters consistently reverses stress-induced downregulation of BDNF/TrkB signaling in the hippocampus and prefrontal cortex across multiple animal models.
- The stress-resilience effects depend on synergistic activation of PI3K/Akt, MAPK/ERK and PLCγ cascades, which collectively drive neurogenesis, dendritic spine remodeling and synaptic protein synthesis, ultimately restoring hippocampal negative feedback control over the HPA axis.

**What are the implications of the main findings?**

- These findings identify the BDNF/TrkB pathway as a critical molecular hub that translates structured auditory stimulation into systemic stress adaptation, offering a mechanistic rationale for music-based interventions in animal welfare and biomedical research.
- The delineation of effective acoustic parameters provides a framework for developing standardized, non-invasive auditory enrichment strategies and underscores the urgent need for systematic parametric studies to guide cross-species translation.

**Abstract:**

**Background/Objectives**: Chronic stress poses a major threat to animal health and welfare, yet current interventions are often invasive, poorly compliant, or lack long-term feasibility. This narrative review synthesizes preclinical mechanistic evidence from mammalian animal models to examine how structured auditory stimulation engages neurotrophic signaling. Structured light music—an auditory stimulus with defined acoustic parameters (e.g., tempo, intensity, and spectral frequency)—has emerged as a promising non-invasive strategy for promoting stress adaptation. However, the molecular mechanism of its effects remains unclear, and systematic analyses linking acoustic features to signaling pathways are scarce. Brain-derived neurotrophic factor (BDNF) and its high-affinity receptor tropomyosin receptor kinase B (TrkB) constitute a central signaling axis regulating neuroplasticity and stress adaptation, suggesting that this pathway may serve as a critical molecular interface linking auditory stimulation to stress-regulatory processes. **Methods**: In this review, we focus on structured light music stimuli with distinct acoustic characteristics and examine the molecular mechanisms by which they regulate neural plasticity through the BDNF/TrkB pathway. **Results:** Existing studies have shown that light music is associated with reversal of stress-induced down-regulation of BDNF/TrkB signaling in the hippocampus and prefrontal cortex. This effect is accompanied by enhanced neurogenesis, dendritic spine remodeling, and synaptic protein synthesis, which are linked to the coordinated activation of three downstream pathways: PI3K/Akt, MAPK/ERK and PLCγ. This cascade reaction further correlates with restoration of negative feedback inhibition of the hypothalamic–pituitary–adrenal axis in the hippocampus, reduces the level of glucocorticoids, and alleviates oxidative stress and inflammatory damage. **Conclusions**: This review proposes that the BDNF/TrkB pathway may serve as a key molecular basis for light music to regulate neural plasticity and achieve systemic anti-stress effects, and provides new ideas for animal welfare management and the formulation of standardized acoustic intervention strategies.

## 1. Introduction

Chronic stress represents a pervasive yet underestimated threat to animal health and welfare in intensive farming systems, laboratory environments, and captive settings [1]. Animals maintained under these conditions are frequently exposed to multiple stressors, including overcrowding, transportation, environmental noise, and social conflict [2]. Persistent activation of the hypothalamic–pituitary–adrenal (HPA) axis and sympathetic nervous system in response to these challenges results in excessive glucocorticoid secretion, impaired neuroplasticity, and compromised immune function [3]. Beyond its detrimental effects on animal welfare, chronic stress also reduces production efficiency and compromises the reliability and reproducibility of experimental outcomes [2]. Therefore, identifying safe, effective, and non-invasive approaches for stress management represents an important scientific and translational challenge.

Auditory enrichment has recently attracted increasing attention as a non-pharmacological and non-invasive environmental intervention strategy [4,5]. However, previous reviews have generally considered “music” as a broad and heterogeneous category, focusing primarily on behavioral outcomes rather than the underlying molecular mechanisms [6]. Different studies have applied diverse musical styles, sound intensities, exposure durations, and frequency ranges, yet these interventions are often collectively described as “music therapy” or “music exposure.” This conceptual ambiguity obscures a fundamental mechanistic question: how do structured auditory stimuli with specific acoustic properties engage molecular pathways to regulate stress adaptation?

Brain-derived neurotrophic factor (BDNF) and its high-affinity receptor tropomyosin receptor kinase B (TrkB) represent a core molecular system regulating neuronal survival, synaptic plasticity, and cognitive function within the central nervous system. Through coordinated regulation of gene transcription, protein trafficking, and synaptic transmission, BDNF/TrkB signaling orchestrates neuronal adaptation and plasticity [7,8]. The integrity of this signaling axis is essential not only for maintaining hippocampal plasticity but also for supporting hippocampus-dependent inhibitory feedback regulation of the HPA axis.

Chronic stress disrupts this regulatory system through multiple mechanisms. Sustained glucocorticoid elevation directly suppresses BDNF transcription and interferes with effective TrkB receptor activation, resulting in marked reductions in BDNF/TrkB signaling within the hippocampus and prefrontal cortex [9]. This impairment subsequently weakens hippocampal inhibitory regulation of the HPA axis, thereby promoting persistent neuroendocrine activation [10]. Importantly, recent studies suggest that structured auditory stimulation with defined acoustic characteristics, such as light music, has been reported to enhance BDNF and TrkB expression in stress-exposed animals [11,12,13]. Moreover, the anxiolytic effects induced by auditory stimulation are substantially diminished or abolished in BDNF-deficient or genetically modified mouse models, providing evidence that BDNF signaling may be functionally required for these behavioral benefits [14].

Despite these advances, a comprehensive mechanistic framework explaining how specific acoustic stimulation engages the BDNF/TrkB pathway to produce stress resilience remains lacking. In particular, the relationship between acoustic parameters and biological efficacy, the molecular events linking auditory perception to neuroplastic remodeling, and the downstream signaling cascades responsible for systemic stress regulation remain insufficiently understood.

In this review, we propose an integrated mechanistic framework linking structured light music stimulation to BDNF/TrkB activation, hippocampal plasticity, HPA axis regulation, and peripheral stress adaptation. We discuss the acoustic parameters of light music, examine the role of BDNF/TrkB signaling in stress regulation, and summarize evidence for downstream cascades and neuroplastic remodeling. Finally, we present a mechanistic model that integrates these levels and offer perspectives for standardized acoustic strategies in animal welfare and stress management.

## 2. Literature Search Strategy

Given the narrative scope of this review, we conducted a structured literature search to identify relevant studies investigating the relationship between structured auditory stimulation, BDNF/TrkB signaling, and stress adaptation. The following databases were systematically searched: PubMed, Embase, PsycINFO, and Web of Science, supplemented by manual screening of reference lists and targeted searches in Google Scholar to capture additional relevant publications. The search covered the period from January 2000 to June 2026.

The principal search terms and their Boolean combinations included: (“structured light music” OR “auditory enrichment” OR “music exposure” OR “rhythmic auditory stimulation”) AND (“BDNF” OR “TrkB” OR “neurotrophic factor”) AND (“HPA axis” OR “glucocorticoid” OR “corticosterone” OR “stress resilience”) AND (“neuroplasticity” OR “hippocampus” OR “synaptic plasticity”).

For inclusion, we prioritized original peer-reviewed animal studies (mammalian models, including mice, rats, pigs, and sheep) that reported measurable outcomes related to BDNF/TrkB signaling, hippocampal structural remodeling, or HPA axis regulation in response to acoustic stimuli with defined or inferable physical parameters (frequency, tempo, or intensity). We excluded human clinical trials, case reports, and studies that used music merely as a background condition without molecular or endocrine endpoints.

To distinguish “structured light music” from general auditory enrichment, rhythmic stimulation, or noise, we adopted the following operational criteria: (1) Structured light music was defined as stimuli containing melodic/harmonic organization and falling within the commonly reported acoustic ranges of 200–1000 Hz (dominant spectral energy), 60–80 beats per minute, and 50–60 dB sound pressure level; (2) General auditory enrichment referred to non-musical environmental sounds (e.g., natural sounds or species-specific vocalizations) without defined harmonic structure; (3) Rhythmic stimulation was defined as isolated periodic clicks or beats devoid of melodic content; and (4) Noise was defined as aperiodic or broadband acoustic signals (e.g., white or pink noise) lacking temporal predictability. Studies falling into categories (2)–(4) were included only when they provided direct comparative data against structured music conditions.

## 3. Acoustic Parameters and Biological Effectiveness of Structured Light Music

As a structured auditory stimulus, the reproducibility of the stress-adaptive effects induced by light music largely depends on the precise definition of its acoustic properties. Unlike human music perception, which is strongly influenced by cultural background and aesthetic preferences, the biological effects of music in animal studies require operational definitions based on measurable acoustic parameters, including frequency, tempo, and intensity [4]. Such a parameter-based framework distinguishes structured light music from other types of acoustic stimulation, including rock music (typically characterized by high rhythmic complexity, rapid tempo, broad spectral distribution, and greater intensity fluctuations), atonal music (which lacks predictable rhythmic and harmonic organization), and white noise (which consists of randomly distributed acoustic signals without structured temporal patterns). Establishing these acoustic parameters is therefore essential not only for experimental reproducibility but also for clarifying the causal relationships between specific auditory features and biological outcomes.

### 3.1. Core Acoustic Parameters Underlying the Stress-Resilience Effects of Light Music

In this review, “structured light music” refers specifically to auditory stimuli that possess identifiable melodic and harmonic organization, as opposed to unstructured noise, isolated rhythmic beats, or non-musical environmental sounds. The biological effects of structured light music are governed by the tripartite interaction of frequency, tempo, and intensity. Frequency determines the initial patterns of activation within the auditory pathway; tempo may influence autonomic regulation through rhythmic entrainment mechanisms; and sound intensity determines whether an acoustic stimulus is perceived as a biologically relevant auditory signal or as a potential stressor. Deviations beyond experimentally effective ranges of these parameters may weaken or even reverse the beneficial effects.

#### 3.1.1. Frequency

Current animal studies have primarily focused on the low-to-mid frequency range of 200–1000 Hz, which overlaps with auditory-sensitive regions in many mammalian species [15] and propagates efficiently through air. At the cochlear level, this frequency range activates spatially distinct hair cell populations [16] and may also be detected via vibrotactile pathways [17]. The biological effectiveness of this range arises from a multilevel alignment of physical propagation, peripheral sensitivity, and central representation: low-to-mid frequencies propagate well, overlap with cochlear sensitivity, and reliably activate low-frequency cortical areas [17].

#### 3.1.2. Tempo

A tempo range of 60–80 bpm is commonly used in light music studies [13]. While this range may correspond to resting heart rates in some species (e.g., cattle), the similarity between musical tempo and heart rate does not by itself demonstrate physiological entrainment [18]. Instead, rhythmic perception likely engages auditory–motor integration networks [19] and can induce neural oscillatory entrainment through cortico–basal ganglia–thalamic circuits [20]. The 60–80 bpm range may also interact with low-frequency neural oscillations associated with resting states, contributing to relaxation via autonomic modulation [21]. Thus, tempo-dependent effects likely emerge from dynamic interactions between external rhythmic structures and endogenous neural oscillatory systems, rather than from simple heart-rate matching.

#### 3.1.3. Sound Intensity

The intensity of structured light music is typically maintained between 50 and 60 dB, and generally does not exceed 70 dB [13]. Auditory brainstem response studies indicate that the hearing threshold of Holstein cattle is approximately 90 dB [22], suggesting that 50–60 dB represents a moderate-level auditory stimulus that remains within the detectable range without reaching potentially aversive intensities.

Importantly, excessive sound intensity may itself function as an environmental stressor by activating stress-responsive neural circuits, including the HPA axis, and increasing glucocorticoid secretion. Therefore, appropriate regulation of sound intensity represents an essential component of experimental design, alongside frequency and tempo selection, for optimizing the biological effects of auditory stimulation.

Collectively, structured light music stimulation in animal studies can be operationally described as an organized auditory stimulus frequently characterized by a frequency range of 200–1000 Hz (referring to the dominant spectral energy of the complex acoustic signal), a tempo of 60–80 bpm, and an intensity of 50–60 dB. Importantly, these parameters represent experimentally observed characteristics from available studies rather than a fixed biological definition, and their biological effects likely emerge from interactions among multiple acoustic features rather than from any single parameter alone. These acoustic parameters correspond to distinct aspects of auditory encoding, rhythmic synchronization, and sensory perception, providing a physiological foundation for transforming physical sound signals into biologically meaningful neural responses.

### 3.2. Biological Basis of Acoustic Parameter Perception by the Auditory System

The biological effectiveness of structured light music stimulation is closely associated with the interaction between its acoustic characteristics and the physiological properties of the auditory system.

At the cochlear level, the basilar membrane exhibits a highly organized frequency-dependent spatial representation. High-frequency sounds induce maximal vibration near the basal region, whereas low-frequency sounds preferentially activate the apical region. This tonotopic organization represents a fundamental structural principle of mammalian auditory processing [23,24,25]. Acoustic signals within the 200–1000 Hz range can activate frequency-specific populations of cochlear hair cells, thereby generating temporally structured auditory inputs that are transmitted to central auditory circuits [26].

At the cortical level, auditory neurons exhibit frequency-selective tuning properties, with individual neurons displaying preferential responses to specific frequency ranges [27]. The 200–1000 Hz frequency range encompasses extensive neuronal populations within low-frequency auditory cortical representations and may therefore induce stable and reproducible cortical activation patterns. Rodent studies further demonstrate that repeated auditory stimulation can induce long-lasting plastic changes in auditory cortical frequency maps [28], suggesting that chronic exposure to structured light music stimulation may facilitate experience-dependent auditory plasticity through cortical remodeling mechanisms.

At the level of rhythmic synchronization, external auditory rhythms within the 60–80 bpm range may interact with autonomic regulation through auditory–autonomic coupling pathways [29]. Heart-rate variability studies indicate that rhythmic auditory stimulation can modulate cardiac activity, whereas slow-tempo sounds tend to enhance parasympathetic tone [30].

Together, these auditory-driven autonomic adjustments may contribute to the physiological relaxation responses observed following structured rhythmic stimulation.

## 4. Molecular Mechanisms of Stress Regulation Mediated by the BDNF/TrkB Signaling Pathway

### 4.1. Molecular Architecture of the BDNF/TrkB Signaling Pathway

The BDNF/TrkB signaling pathway represents a central molecular interface linking auditory stimulation to neuroplastic adaptation and stress resilience. This pathway regulates three critical components of stress regulation: neuronal survival, synaptic plasticity, and hippocampal inhibitory control of the HPA axis.

BDNF is one of the most widely expressed neurotrophic factors in the central nervous system and belongs to the neurotrophin family. It plays essential roles in neuronal survival, differentiation, synaptic plasticity, and cognitive regulation [31]. The synthesis and maturation of BDNF involve a highly regulated multistep process. The BDNF gene is transcribed into precursor mRNA, which is translated into pre-proBDNF. Following removal of the signal peptide in the endoplasmic reticulum, pre-proBDNF is converted into proBDNF. This precursor is subsequently transported to the Golgi apparatus, where proteolytic enzymes such as furin cleave it into mature BDNF (mBDNF) [32].

Following maturation, mBDNF is packaged into synaptic vesicles or dense-core vesicles and released into the synaptic cleft in an activity-dependent manner. In addition, extracellular conversion of proBDNF into mBDNF can occur through matrix metalloproteinases (MMP-2/9) or plasmin-dependent cleavage [33]. Together, intracellular processing and extracellular conversion establish a dynamic regulatory system that enables precise spatial and temporal control of BDNF signaling [34,35].

BDNF primarily exerts its biological functions through its high-affinity receptor TrkB [36]. TrkB is a receptor tyrosine kinase consisting of an extracellular ligand-binding domain, a transmembrane region, and an intracellular kinase domain [37]. Binding of mBDNF to the extracellular domain of TrkB induces receptor dimerization and subsequent autophosphorylation of intracellular tyrosine residues [7]. These phosphorylated residues function as docking platforms for downstream signaling adaptors, initiating three canonical intracellular cascades: phosphoinositide 3-kinase/protein kinase B (PI3K/Akt), mitogen-activated protein kinase/extracellular signal-regulated kinase (MAPK/ERK), and phospholipase Cγ (PLCγ) signaling pathways [38].

The PLCγ pathway mediates rapid synaptic responses by hydrolyzing phosphatidylinositol-4,5-bisphosphate into inositol trisphosphate and diacylglycerol. Inositol-1,4,5-trisphosphate induces intracellular calcium release, activating calcium/calmodulin-dependent protein kinases and the transcription factor cAMP response element-binding protein, thereby regulating immediate early gene expression.

The PI3K/Akt pathway primarily promotes neuronal survival. Activation of Akt suppresses apoptotic signaling through phosphorylation of pro-apoptotic proteins such as Bad, thereby protecting neurons against mitochondrial apoptosis [39,40,41].

The MAPK/ERK pathway primarily mediates long-term structural and functional plasticity. Through activation of the Ras–Raf–MEK–ERK cascade, BDNF/TrkB signaling promotes transcriptional activation and synaptic protein synthesis, thereby facilitating dendritic remodeling and synaptic adaptation [42].

Together, these three signaling cascades operate in a coordinated temporal and spatial manner, forming a hierarchical regulatory network that supports rapid synaptic modulation, intermediate neuronal maintenance, and long-term structural remodeling.

Within the hippocampus, both BDNF and TrkB are highly expressed, with the dentate gyrus and CA3 subregions representing areas with particularly enriched BDNF/TrkB signaling activity [37]. This signaling axis plays a pivotal role in regulating hippocampal neurogenesis and synaptic plasticity. On one hand, BDNF promotes the proliferation and differentiation of hippocampal neural stem cells, while facilitating dendritic growth and dendritic spine maturation of newly generated neurons [43]. However, BDNF is not the sole regulator of hippocampal neurogenesis; other hormones—including insulin-like growth factor-1 (IGF-1), estradiol, and testosterone—also contribute to the proliferation, differentiation, and survival of newly generated neurons. On the other hand, BDNF regulates long-term potentiation and long-term depression through TrkB-dependent synaptic protein synthesis, thereby establishing the molecular foundation underlying learning and memory formation [43].

**Figure 1:**
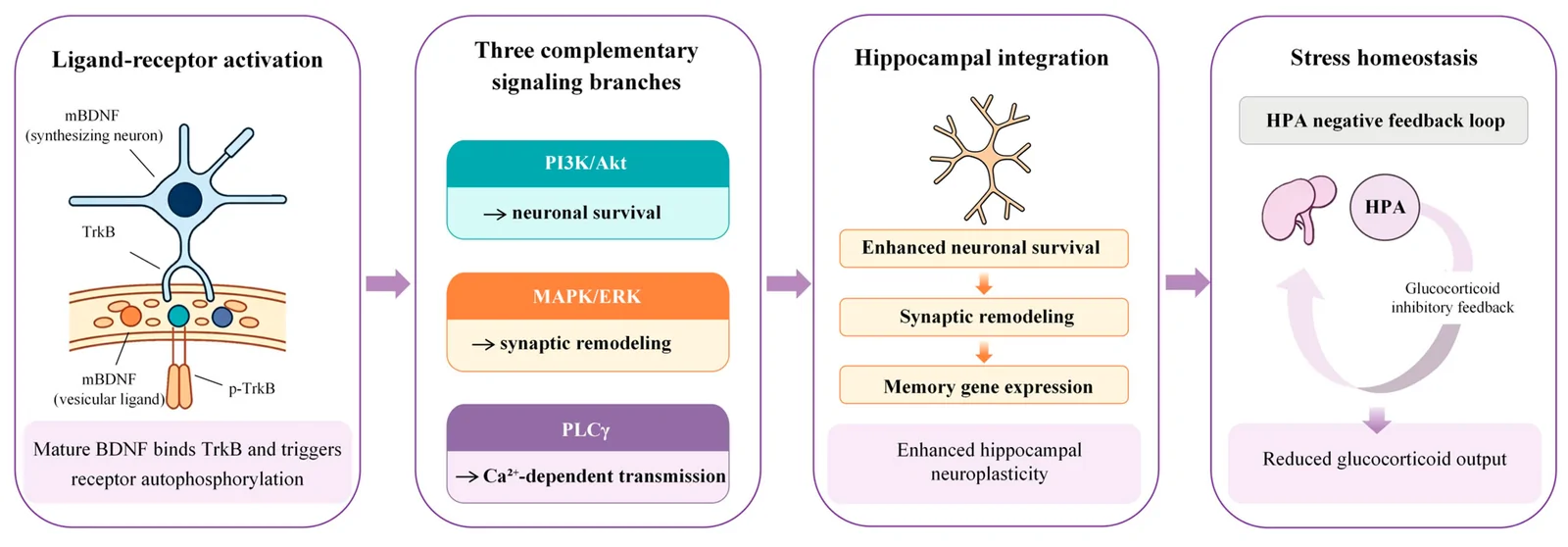
Simplified molecular architecture of the BDNF/TrkB signaling pathway. Binding of mature BDNF to the TrkB receptor induces receptor dimerization and autophosphorylation, leading to the coordinated activation of PI3K/Akt, MAPK/ERK, and PLCγ signaling cascades. The PI3K/Akt branch promotes neuronal survival, the MAPK/ERK branch mediates synaptic protein expression and dendritic spine remodeling, and the PLCγ branch regulates calcium-dependent synaptic transmission. These complementary pathways converge to enhance hippocampal neuroplasticity, reinforce HPA axis negative feedback, and restore stress homeostasis.

As a critical brain region responsible for inhibitory feedback regulation of the HPA axis, the functional integrity of hippocampal BDNF/TrkB signaling is essential for maintaining appropriate stress responsiveness and preventing excessive neuroendocrine activation [44]. Following BDNF transcription, maturation, and activity-dependent release, mature BDNF activates TrkB receptors and engages downstream PI3K/Akt, MAPK/ERK, and PLCγ cascades. Through coordinated regulation of neuronal survival, synaptic remodeling, and hippocampal circuit integrity, BDNF/TrkB signaling contributes to restoration of HPA axis homeostasis and represents a key molecular substrate underlying stress resilience. A comprehensive schematic representation of this molecular regulatory framework is presented in Figure 1.

### 4.2. Chronic Stress-Induced Suppression of the BDNF/TrkB Signaling Pathway

Chronic stress suppresses BDNF/TrkB signaling through multiple convergent molecular mechanisms. Dysregulation of this pathway represents a critical molecular interface linking prolonged stress exposure with hippocampal neuroplasticity impairment and maladaptive HPA axis regulation.

Among these mechanisms, glucocorticoid-mediated inhibition of BDNF transcription represents one of the best-characterized pathways. Chronic stress activates the HPA axis, resulting in sustained elevation of glucocorticoid levels. Excessive glucocorticoids bind to glucocorticoid receptors expressed in hippocampal neurons and directly interact with regulatory regions of the BDNF gene, particularly promoter regions II and IV, thereby suppressing BDNF transcription [45]. Consistent with this mechanism, animal studies have demonstrated that chronic restraint stress significantly reduces hippocampal BDNF mRNA expression, and this reduction is negatively correlated with persistent elevation of circulating glucocorticoids [46].

In addition to reducing BDNF ligand availability, chronic stress also disrupts signal transmission by interfering with TrkB receptor function. Glucocorticoid receptors can physically interact with TrkB receptors, thereby impairing effective BDNF–TrkB coupling and reducing receptor activation efficiency [47]. Increasing evidence indicates a close bidirectional interaction between BDNF/TrkB signaling and the glucocorticoid/glucocorticoid receptor system within the central nervous system [48]. Therefore, chronic stress produces a dual-level disruption of BDNF/TrkB signaling by simultaneously reducing ligand availability and compromising receptor responsiveness.

Downregulation of BDNF/TrkB signaling contributes to the establishment of a self-reinforcing pathological loop involving hippocampal structural deterioration and impaired HPA axis feedback regulation [49]. On one hand, reduced hippocampal BDNF signaling directly compromises neurogenesis and synaptic maintenance. Studies using TrkB-deficient models further demonstrate that loss of TrkB signaling in newly generated hippocampal neurons impairs dendritic growth, reduces dendritic spine density, and disrupts functional neuronal integration [50].

Conversely, hippocampal structural and functional impairment weakens its inhibitory control over HPA axis activation. Under physiological conditions, the hippocampus restrains corticotropin-releasing hormone neurons in the hypothalamic paraventricular nucleus via extensive glutamatergic projections [51]. When hippocampal BDNF/TrkB signaling is compromised, this inhibitory mechanism is attenuated, leading to sustained activation of paraventricular corticotropin-releasing hormone neurons and elevated adrenocorticotropic hormone and glucocorticoid secretion [44].

Elevated glucocorticoids further suppress BDNF transcription [45], thereby reinforcing a self-amplifying pathological cycle: chronic stress elevates glucocorticoids, excessive glucocorticoids inhibit BDNF/TrkB signaling, impaired BDNF signaling disrupts hippocampal function, and weakened hippocampal inhibition further enhances HPA axis activation [48].

**Figure 2:**
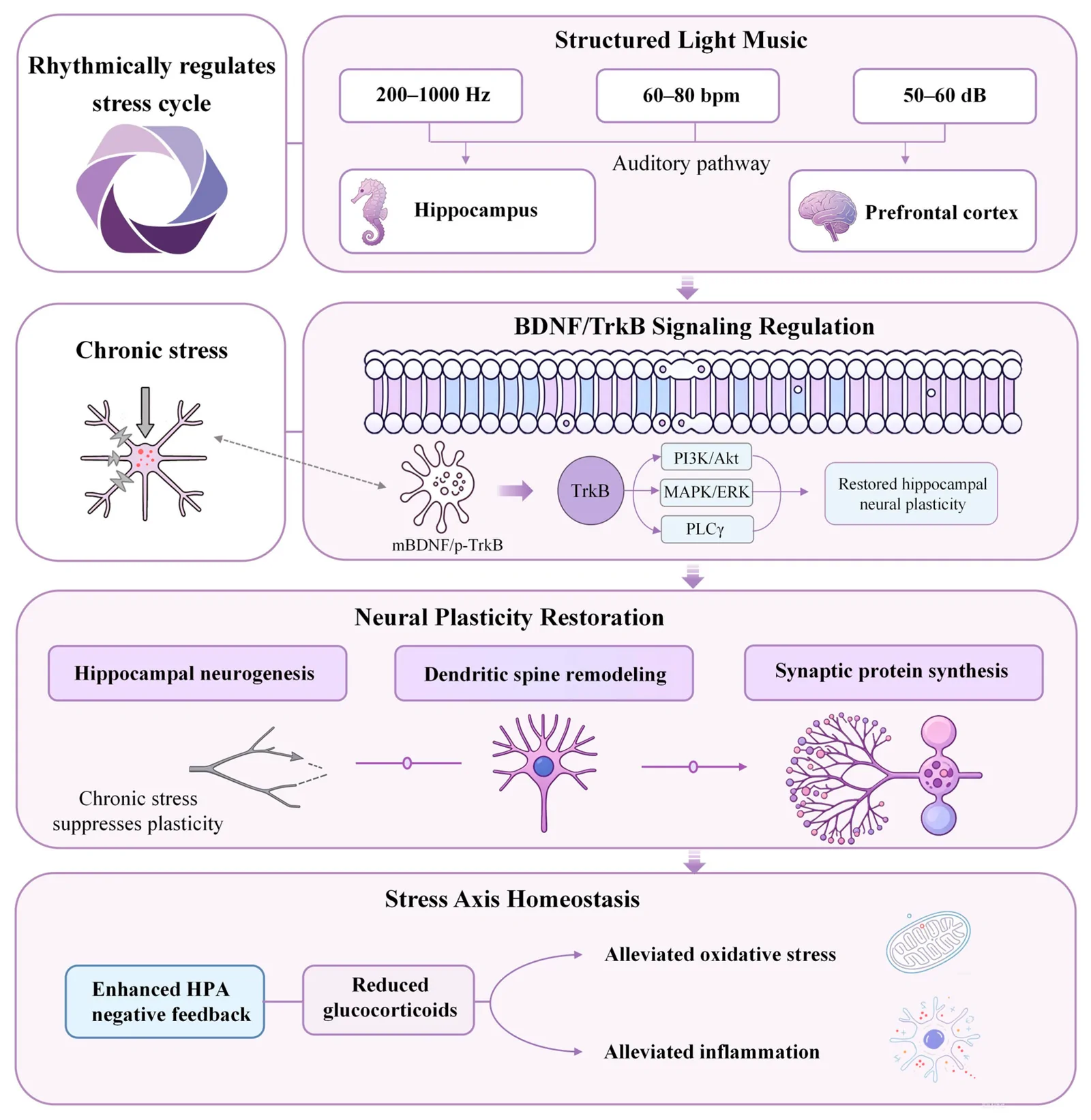
Minimal model of the chronic stress vicious cycle mediated by glucocorticoid suppression of BDNF/TrkB signaling. Chronic stress elevates circulating glucocorticoid levels, which reduce BDNF transcription and inhibit TrkB signaling, leading to downregulated BDNF/TrkB activity and hippocampal dysfunction. Impaired hippocampal control of the HPA axis weakens negative feedback restraint, further increasing glucocorticoid secretion and reinforcing a self-amplifying stress loop.

Collectively, chronic stress suppresses BDNF/TrkB signaling through glucocorticoid–glucocorticoid receptor-dependent mechanisms, leading to impaired hippocampal neuroplasticity, disrupted inhibitory regulation of the HPA axis, and progressive enhancement of stress vulnerability. This glucocorticoid-driven BDNF/TrkB suppression cycle provides a mechanistic framework for understanding how chronic stress produces persistent neural and endocrine dysfunction, and establishes the biological rationale for interventions capable of restoring BDNF-dependent neuroplasticity. The stress-induced pathological cycle is illustrated in Figure 2.

## 5. Activation of the BDNF/TrkB Signaling Pathway by Structured Light Music

Although numerous behavioral studies have demonstrated that structured light music can alleviate anxiety-like behaviors and improve stress-related outcomes, the molecular cascade linking auditory perception to neuroprotective adaptation remains poorly defined. Previous investigations have largely focused on systemic physiological indicators, whereas the contribution of the BDNF/TrkB neurotrophic signaling axis as a potential molecular mediator of auditory-induced stress resilience has not been systematically synthesized. Here, we review the activation of BDNF/TrkB signaling by structured light music from three angles: molecular regulation of the pathway, stimulus specificity, and genetic validation of its biological necessity.

### 5.1. Activation and Restoration of BDNF/TrkB Signaling by Light Music

Structured light music has been shown to be associated with activation of BDNF/TrkB signaling across three experimentally distinct paradigms, each confirming the reproducibility and biological relevance of this response.

In a post-stress intervention model, Cheng et al. systematically compared the effects of light music, classical music, atonal music, and rock music on depression-like behaviors induced by chronic unpredictable mild stress (CUMS) in mice [13]. At the behavioral level, only light music and classical music significantly increased sucrose preference and reduced immobility time in the tail suspension test, whereas atonal music and rock music failed to produce antidepressant-like effects. At the molecular level, exposure to light music or classical music was accompanied by reduced serum corticosterone levels, restoration of hippocampal glucocorticoid receptor expression, and re-establishment of BDNF-related signaling activity. These molecular changes were further associated with increased expression of synaptic proteins and enhanced neurogenesis-related markers [11,12,13]. Collectively, these findings indicate that the biological effects of music are not driven simply by the presence of sound itself, but depend on specific structural characteristics of acoustic stimulation.

In a stress-synchronized preventive model, Fu et al. investigated whether simultaneous music exposure during CUMS induction could prevent stress-related pathological changes. Under this experimental paradigm, music exposure effectively prevented the development of CUMS-induced depressive- and anxiety-like behaviors and normalized serum corticosterone levels. At the molecular level, music exposure reversed stress-induced reductions in BDNF and B-cell lymphoma-2 (Bcl-2) expression, while simultaneously suppressing oxidative stress and inflammatory responses [11]. These findings suggest that BDNF/TrkB activation may represent an early adaptive molecular event underlying the stress-buffering effects of structured auditory stimulation.

In a normal developmental model, Wang et al. examined the effects of different tonal music stimuli on neuronal structural plasticity in the hippocampus and prefrontal cortex of juvenile mice. Following 14 consecutive days of music exposure (2 h/day), mice exposed to music exhibited significantly increased neuronal numbers, dendritic spine numbers, and spine density per unit dendritic length compared with controls. Furthermore, expression levels of synaptic plasticity-related proteins, including synaptophysin (SYP) and postsynaptic density protein-95, were markedly elevated. At the molecular level, music exposure significantly enhanced the expression of BDNF, TrkB, cAMP response element-binding protein, and key components of the PI3K/Akt and MAPK/ERK signaling pathways in the hippocampus and prefrontal cortex [11].

Across preventive and therapeutic regimens, as well as in both stress-exposed and normally developing animals, structured light music consistently enhances BDNF/TrkB signaling and its downstream molecular cascades. These convergent observations establish the BDNF/TrkB axis as a key molecular interface that translates structured auditory stimulation into neuroplastic adaptation and stress resilience.

### 5.2. Stimulus Specificity of Light Music-Induced BDNF/TrkB Activation

The stress-resilience effects induced by structured light music exhibit distinct stimulus specificity, which emerges at both acoustic and neuroanatomical levels. At the acoustic level, different categories of music produce differential biological outcomes, whereas at the neural level, BDNF/TrkB activation occurs selectively within specific brain regions associated with cognitive and emotional regulation.

Regarding music-type specificity, Cheng et al. compared four categories of music under identical experimental conditions and demonstrated that only light music and classical music significantly alleviated CUMS-induced depression-like behaviors and enhanced BDNF signaling, whereas atonal music and rock music failed to produce comparable benefits [13]. These findings indicate that the biological effects of music are not intrinsic properties of acoustic stimulation per se, but instead depend on the presence of specific structural features within the auditory signal.

The inability of rock music and atonal music to activate BDNF signaling may be explained by their distinct acoustic characteristics. Rock music is typically associated with higher sound intensity (often exceeding 85 dB) and faster tempo (>120 bpm), characteristics that may increase auditory arousal and potentially engage stress-responsive neuroendocrine pathways, including HPA axis activation and glucocorticoid secretion. Such responses could counteract potential neuroprotective effects. In contrast, atonal music lacks predictable rhythmic and harmonic organization and may therefore fail to effectively induce endogenous neural oscillatory entrainment. Together, these observations suggest that there appears to be an important factor associated with recruitment of BDNF/TrkB-related neuroplastic responses.

Regarding regional specificity, Xing et al. investigated the effects of Mozart’s K.448 sonata exposure on spatial learning in developing rats. Early-life music exposure significantly improved performance in the Morris water maze. At the molecular level, music exposure selectively increased BDNF/TrkB expression in the dorsal hippocampal CA3 region and dentate gyrus, whereas the CA1 region showed no significant changes [52].

This regional selectivity may reflect functional specialization among hippocampal subregions. The CA3 region plays a critical role in pattern completion and retrieval of spatial memories, whereas the dentate gyrus contributes to pattern separation and encoding of new memories. Therefore, selective enhancement of BDNF signaling within CA3 and dentate gyrus suggests that structured music stimulation may preferentially facilitate hippocampal circuits involved in spatial learning and memory consolidation.

### 5.3. Genetic Evidence Supporting BDNF as a Necessary Mediator

Previous correlational studies have linked structured light music exposure to BDNF/TrkB pathway activation, yet whether this signaling is mechanistically required for music-induced stress resilience remains unresolved. Specifically, BDNF activation could be either an incidental correlate or an essential mediator of the behavioral effects. Genetic loss- and gain-of-function experiments have now begun to settle this question by establishing a causal role for the pathway.

To determine whether BDNF signaling is necessary for the anxiolytic effects of music, Li et al. employed two genetically modified mouse models: humanized BDNF Val66Met (Met/Met) knock-in mice and BDNF heterozygous knockout mice [14]. The BDNF Val66Met polymorphism is a common functional single-nucleotide variation associated with increased susceptibility to anxiety disorders and depression.

Both mouse strains were exposed to music or white noise, and anxiety-like behaviors were evaluated using the open-field test and elevated plus maze. The results demonstrated that music exposure significantly reduced anxiety-like behaviors in BDNF Met/Met mice, whereas comparable behavioral benefits were not observed in BDNF heterozygous knockout mice. Mechanistically, this genotype-dependent difference was associated with distinct BDNF responses to music stimulation. Music exposure significantly increased BDNF expression in the prefrontal cortex, amygdala, and hippocampus of BDNF Met/Met mice, whereas BDNF-deficient mice failed to exhibit comparable transcriptional upregulation following auditory stimulation [14].

The parallel alterations observed at behavioral and molecular levels provide strong support for the concept that music-induced enhancement of BDNF signaling is not simply an epiphenomenon accompanying auditory exposure, but rather represents a functional molecular requirement underlying its anxiolytic actions. Further studies have demonstrated that BDNF Val66Met knock-in mice exhibit increased anxiety-related behaviors under stressful conditions and reduced sensitivity to antidepressant treatment with fluoxetine [53]. Together, these findings suggest that music-based interventions may have particular relevance for individuals carrying BDNF Met variants, who exhibit altered neuroplasticity and stress susceptibility [14].

Evidence for structured light music-induced BDNF/TrkB pathway activation has converged across three dimensions: (1) molecular regulation, including transcriptional and protein-level enhancement of BDNF/TrkB signaling; (2) stimulus specificity, demonstrated by selective responses to particular music structures and brain regions; and (3) genetic causality, established through BDNF knock-in and knockout models. However, an important mechanistic question remains unresolved: how does enhanced BDNF/TrkB signaling translate into functional neuroplastic remodeling? Specifically, whether the three major downstream cascades—PI3K/Akt, MAPK/ERK, and PLCγ—are coordinately activated by structured light music, and how each pathway contributes to stress resilience, remain critical areas for future investigation.

## 6. Downstream Signaling Cascades and Neuroplastic Remodeling Following BDNF/TrkB Activation

Following BDNF binding to the TrkB receptor, multiple intracellular signaling cascades are activated to regulate neuronal plasticity. These downstream pathways do not function as isolated signaling modules; instead, they operate as an integrated molecular network that coordinates neuronal survival, synaptic adaptation, and structural remodeling [54,55]. Structured light music has been associated with engagement of these downstream cascades and with reversal of chronic stress-induced hippocampal structural deficits.

### 6.1. Coordinated Activation of Downstream Signaling Pathways and Functional Outputs

Upon BDNF binding to TrkB, phosphorylated tyrosine residues within the intracellular domain of TrkB recruit multiple adaptor proteins and initiate parallel activation of three major intracellular signaling cascades. Through this coordinated signaling architecture, BDNF regulates neuronal survival, functional maintenance, and structural remodeling across distinct temporal scales. This canonical signaling framework provides a biologically plausible basis for understanding how structured auditory stimulation might exert neuroprotective effects; however, direct evidence for the coordinated engagement of all three pathways specifically in the context of music-induced stress resilience remains limited.

#### 6.1.1. PI3K/Akt Signaling Pathway: Regulation of Neuronal Survival and Metabolic Stability

The PI3K/Akt pathway represents a primary downstream effector pathway mediating BDNF-dependent neuronal survival. However, this pathway is not exclusively activated by BDNF; other signaling molecules—including growth hormone (GH), insulin-like growth factor-1 (IGF-1), and nutrients—can also engage PI3K/Akt signaling in various cellular contexts. Following TrkB activation, BDNF induces PI3K recruitment and activation, which subsequently promotes phosphorylation of Akt. Activated Akt inhibits mitochondrial apoptotic signaling through phosphorylation of downstream pro-apoptotic targets, including Bad and caspase-9, thereby enhancing neuronal resilience against stress-induced apoptotic damage [39].

In addition, Akt activation stimulates the mammalian target of rapamycin (mTOR) pathway, promoting local synthesis of synaptic proteins and providing essential molecular substrates for synaptic remodeling [41]. Consistent with this mechanism, animal studies have demonstrated that music exposure increases PI3K and Akt expression levels in the hippocampus and prefrontal cortex of developing mice [12]. These findings indicate that PI3K/Akt signaling may represent a key molecular mechanism potentially contributing to the preservation of neuronal integrity and counteracts stress-induced neural vulnerability.

#### 6.1.2. MAPK/ERK Signaling Pathway: Regulation of Long-Term Synaptic Plasticity

The MAPK/ERK pathway primarily mediates the long-lasting synaptic remodeling effects of BDNF signaling. Following BDNF–TrkB interaction, the Ras–Raf–MEK–ERK cascade is activated. Phosphorylated ERK subsequently translocates into the nucleus, where it promotes activation of transcription factors including cAMP response element-binding protein. This process enhances transcription of genes encoding synaptic structural proteins, such as synaptophysin (SYP), postsynaptic density protein-95, and dendritic spine-associated proteins [42]. In mouse models, tonal music stimulation enhances ERK expression, along with dendritic spine density and synaptic protein levels [12]. These observations support the concept that MAPK/ERK signaling serves as a molecular bridge connecting structured auditory stimulation with long-term remodeling of neural circuits.

#### 6.1.3. PLCγ Signaling Pathway: Regulation of Rapid Synaptic Responses

The PLCγ pathway primarily mediates the rapid synaptic effects triggered by BDNF/TrkB signaling. Activated PLCγ hydrolyzes membrane phospholipids to generate diacylglycerol and inositol-1,4,5-trisphosphate. Inositol-1,4,5-trisphosphate subsequently induces Ca^2+^ release from intracellular calcium stores, leading to activation of Ca^2+^/calmodulin-dependent protein kinases and cAMP response element-binding protein-dependent transcriptional regulation [55].

Music exposure has been shown to increase the expression of PLCγ1 and downstream molecules, including diacylglycerol and protein kinase C, in the mouse brain [12]. These findings suggest that PLCγ signaling may contribute to the rapid adjustment of synaptic transmission efficiency. Whether this pathway represents a direct mediator of the emotional regulatory effects of structured music stimulation, however, requires further investigation, as PLCγ expression changes have been observed primarily in developing mice without stress exposure.

Together, PLCγ, PI3K/Akt, and MAPK/ERK form a coordinated signaling network supporting rapid synaptic modulation, neuronal survival, and long-term structural remodeling, respectively. While this tripartite signaling architecture is well established in BDNF/TrkB biochemistry, the coordinated engagement of all three pathways specifically in response to structured light music remains largely inferred from studies that examined only subsets of these cascades. The available evidence is consistent with the hypothesis that structured light music exposure may engage these pathways to promote hippocampal plasticity and stress resilience, but direct experimental validation—particularly through simultaneous measurement of all three pathways within the same experimental paradigm—is needed to establish causal relationships.

### 6.2. Light Music-Induced Structural Remodeling and Its Significance for Stress Resilience

One of the major neuropathological hallmarks of chronic stress is the impairment of hippocampal neurogenesis and progressive loss of dendritic spines, which contribute to disrupted synaptic connectivity and compromised stress adaptation [56,57]. Through coordinated engagement of BDNF/TrkB downstream signaling cascades, structured light music may counteract these stress-induced structural abnormalities and facilitate hippocampal circuit remodeling.

First, structured light music exposure facilitates the restoration of hippocampal dendritic spine density. In a maternal separation stress model, music exposure reversed stress-induced reductions in dendritic spine density within the hippocampal CA1 region and was accompanied by improvements in emotional behaviors [52]. These findings suggest that music-induced activation of BDNF signaling may contribute to the recovery of synaptic connectivity disrupted by early-life stress exposure.

Second, structured light music upregulates the expression of key synaptic structural proteins. In developing mice, light music exposure significantly increased levels of synaptophysin (SYP) and postsynaptic density protein-95 in the hippocampus and prefrontal cortex [12]. These synaptic proteins serve as essential molecular substrates for synapse formation, stabilization, and functional maturation, thereby supporting the reconstruction of neuronal communication networks.

Third, structured light music may stimulate hippocampal neurogenic processes. In a rat model exhibiting autism-like behaviors, music exposure increased the number of newborn neurons positive for neurogenesis markers, including BrdU and doublecortin (DCX), within the hippocampal dentate gyrus. This neurogenic effect was accompanied by increased expression of BDNF and TrkB [52]. These observations indicate that BDNF/TrkB signaling may represent a key molecular mechanism through which auditory enrichment supports hippocampal cellular renewal and adaptive plasticity.

Thus, BDNF/TrkB-dependent hippocampal remodeling—at both synaptic and cellular levels—provides a mechanistic link between structured auditory stimulation and systemic stress resilience, likely by restoring the hippocampal structural foundation required for effective HPA axis feedback regulation.

## 7. HPA Axis Regulation and Peripheral Effects as a Proposed Integrative Pathway Involving BDNF/TrkB Signaling

Reduced glucocorticoid levels after music exposure do not, by themselves, prove the complete BDNF/TrkB–hippocampus–HPA axis sequence. Alternative or parallel mechanisms may also contribute, including: (1) direct auditory–hypothalamic projections; (2) amygdala and limbic circuits; (3) autonomic nervous system modulation; and (4) other neurochemical systems (e.g., dopaminergic, serotonergic, noradrenergic, and opioidergic). The following discussion focuses on the BDNF/TrkB–hippocampus axis as one plausible pathway, while acknowledging that multiple mechanisms likely converge to produce the integrated stress-regulatory response.

The HPA axis represents the principal neuroendocrine system responsible for coordinating adaptive stress responses. As described above, chronic stress suppresses hippocampal BDNF/TrkB signaling and compromises hippocampal structural and functional integrity, thereby impairing hippocampus-dependent negative feedback control of HPA axis activity. Based on this mechanistic framework, restoration of hippocampal structure and function through BDNF/TrkB pathway activation by structured light music would be expected to facilitate recovery of inhibitory regulation over HPA axis hyperactivation and attenuate persistent glucocorticoid elevation. This hypothesis is supported across three interrelated domains: neuroendocrine regulation, oxidative stress modulation, and inflammatory control. Together, these findings reveal a hierarchical regulatory cascade extending from central neuroplastic restoration to peripheral systemic homeostasis.

### 7.1. Hippocampal Structural Restoration and Reconstruction of HPA Axis Negative Feedback

The hippocampus represents a critical neural substrate mediating negative feedback regulation of the HPA axis, and its structural integrity and functional state largely determine the intensity and duration of stress-induced neuroendocrine activation. Through engagement of BDNF/TrkB signaling, structured light music promotes hippocampal neurogenesis, dendritic spine remodeling, and synaptic protein synthesis, thereby providing the neuroanatomical foundation required for re-establishing impaired HPA axis feedback regulation. Following hippocampal structural remodeling, restored hippocampal circuitry may recover its inhibitory influence over the hypothalamic paraventricular nucleus. Mechanistically, hippocampal regulation of the HPA axis is mediated through a disynaptic pathway via the bed nucleus of the stria terminalis, which provides inhibitory control over paraventricular corticotropin-releasing hormone neurons [58].

Consistent with this mechanism, studies using CUMS mouse models have demonstrated that light music and classical music exposure are associated with reduced serum corticosterone levels and restoration of hippocampal glucocorticoid receptor expression [13]. Furthermore, when music exposure was administered concurrently with CUMS induction, music intervention was associated with prevention of stress-associated corticosterone elevation and maintained circulating glucocorticoid levels close to those observed in control animals [11]. Similar reductions in circulating glucocorticoids following music exposure have been observed in rat models of stress-induced asthma and piglet models of transportation stress [5]. Across diverse stress paradigms and animal models, these findings are consistent with the proposed pathway in which BDNF/TrkB activation facilitates recovery of HPA axis negative feedback.

### 7.2. Peripheral Effects and Their Potential Links to HPA Axis Normalization

Normalization of HPA axis activity likely represents one major route linking central neuroplastic recovery to peripheral adaptation via neuroendocrine–immune interactions. However, the peripheral anti-inflammatory and antioxidant effects observed after music exposure may also involve autonomic modulation and other neurochemical pathways, rather than HPA normalization alone. Under chronic stress conditions, sustained elevation of glucocorticoids can induce glucocorticoid receptor dysfunction, thereby weakening the inhibitory feedback normally exerted by glucocorticoid signaling on nuclear factor-κB-mediated inflammatory pathways. This maladaptive process promotes excessive production of pro-inflammatory cytokines and increases reactive oxygen species generation [56]. Structured light music restores glucocorticoid homeostasis through the BDNF/TrkB–hippocampus–HPA axis. This cascade in turn attenuates the major drivers of inflammation and oxidative damage, promoting the recovery of systemic stress homeostasis.

#### 7.2.1. Regulation of Inflammatory Responses

In CUMS models, music exposure attenuates inflammatory activation and suppresses the TLR4/NF-κB pathway, shifting cytokine balance from pro-inflammatory (TNF-α, IL-1β, IL-6) to anti-inflammatory (IL-10) [11,59]. Similar effects have been observed in the amygdala of piglets [60]. Collectively, these findings are consistent with the proposed hypothesis that the peripheral anti-inflammatory effects associated with structured music exposure may arise, at least partially, from normalization of neuroendocrine stress signaling, although direct modulation of immune pathways by other mechanisms cannot be excluded.

#### 7.2.2. Regulation of Oxidative Stress

Music exposure also alleviates oxidative stress by modulating the Nrf2 antioxidant pathway, reducing reactive oxygen species production in stress models [11,61]. Collectively, the available evidence is consistent with a proposed integrative pathway in which BDNF/TrkB signaling contributes to hippocampal structural plasticity and HPA axis regulation at the central level, potentially coupled with peripheral modulation of inflammatory and oxidative stress responses. However, this multilevel framework remains largely biologically plausible rather than experimentally established; direct evidence linking each step—from auditory stimulation through central BDNF/TrkB activation, HPA axis normalization, to peripheral anti-inflammatory and antioxidant effects—is currently limited. Future studies are needed to validate this proposed axis and to distinguish HPA-dependent from HPA-independent contributions to peripheral stress adaptation.

## 8. Challenges and Future Perspectives

Although substantial progress has been made in elucidating the stress-resilience effects of structured light music through the BDNF/TrkB signaling pathway, several critical challenges remain unresolved. Addressing these issues will be essential for advancing this field from descriptive observations toward mechanistically defined and clinically translatable auditory interventions.

First, the lack of standardized acoustic definitions represents a major limitation in current studies. Diverse musical stimuli with highly variable acoustic properties are frequently categorized simply as “music exposure” or “music therapy,” making cross-study comparison and reproducibility difficult. Future investigations should establish standardized reporting frameworks incorporating key acoustic parameters, including frequency, tempo, intensity, harmonic structure, and temporal organization. Systematic manipulation of these parameters through factorial experimental designs will help identify the acoustic features that are biologically relevant for activating neuroprotective pathways.

Second, the current literature has focused predominantly on frequency, tempo, and intensity, whereas other potentially important acoustic features—including harmonic structure, spectral composition, rhythmic regularity, timbre, dynamics, predictability, and duration of exposure—remain substantially undercharacterized in relation to BDNF/TrkB signaling and stress adaptation. These parameters are rarely reported in the original studies we reviewed, which limits our ability to draw definitive conclusions about their individual contributions. Future studies employing systematic parametric manipulations are essential to dissect the causal relationships between specific acoustic features and their neurobiological outcomes. This limitation is particularly relevant for cross-study comparisons and for the development of standardized acoustic intervention protocols.

Third, the causal relationship between auditory stimulation, BDNF/TrkB activation, and stress regulation remains incompletely established. Although music exposure has been consistently associated with enhanced BDNF signaling, direct evidence for its necessity is lacking. Moreover, even if BDNF/TrkB is required, whether the three downstream pathways—PI3K/Akt, MAPK/ERK, and PLCγ—are co-engaged or serve distinct functions remains unknown, as most studies have examined only subsets of these cascades in isolation. Future studies integrating pharmacological or genetic pathway manipulations with simultaneous readouts of multiple endpoints are needed to dissect the relative contribution of each cascade and to determine how auditory circuits regulate BDNF expression and downstream hippocampal remodeling.

Fourth, individual variability in responsiveness to auditory stimulation requires greater consideration. Genetic background, developmental stage, sex, stress susceptibility, and baseline neurobiological status may influence the effectiveness of music-based interventions. Future research should identify biological predictors of responsiveness and develop personalized auditory strategies tailored to specific populations and species. In addition, emerging single-cell and spatial transcriptomic approaches may provide new insights into cell-type-specific responses to structured auditory stimulation, revealing previously unrecognized molecular mechanisms.

Fifth, the cross-species generalizability of the findings reviewed here requires careful consideration. The studies included in this review span multiple mammalian species—mice, rats, pigs, sheep, and dogs—that differ markedly in auditory frequency range, cardiovascular physiology, stress neurobiology, and cognitive processing. While the BDNF/TrkB signaling pathway is evolutionarily conserved and its core molecular architecture is similar across mammals, the upstream sensory processing of auditory stimuli and the downstream neuroendocrine outputs are subject to substantial species-specific modulation. For instance, the acoustic parameters that are effective in rodents (e.g., 200–1000 Hz dominant spectral energy) may not directly translate to species with different cochlear tuning or auditory cortical organization. Similarly, the tempo-dependent effects observed in one species cannot be assumed to operate through identical mechanisms in another, given the wide interspecies variation in resting heart rate and autonomic regulation. Therefore, while the BDNF/TrkB–hippocampus–HPA axis framework provides a useful mechanistic hypothesis, its applicability across species must be validated through direct comparative studies rather than inferred from aggregated evidence.

Finally, the predominantly preclinical nature of the evidence reviewed here warrants caution when translating these findings to humans. Unlike animal models, human responses to music are profoundly influenced by personal preferences, familiarity, expectations, emotional context, autobiographical memory, cultural background, and reward processing. Consequently, the acoustic parameters discussed in this review (e.g., 200–1000 Hz, 60–80 bpm, 50–60 dB) cannot currently be considered validated prescriptions for human applications, and extrapolation from animal data to human populations should be made with explicit acknowledgment of these differences. Moreover, while this review focuses on passive music exposure, active musical training—i.e., long-term instrument practice—is known to induce robust structural and functional plasticity in auditory, sensorimotor, and frontotemporal cortical regions, with emerging evidence suggesting BDNF-dependent mechanisms. Future research bridging passive exposure in animal models with active training in humans will help determine whether common BDNF/TrkB-mediated mechanisms underlie both forms of music-induced neuroplasticity.

Overall, structured light music represents a non-invasive, low-cost, and scalable auditory enrichment strategy with potential applications in both biomedical and animal welfare contexts. Future mechanistic studies integrating acoustic science, molecular neuroscience, and systems biology will be essential to define how auditory environments regulate stress adaptation. Such advances may ultimately facilitate the translation of structured auditory interventions from experimental models to clinical applications and sustainable animal management.

## Data Availability

No new data were created or analyzed in this study. Data sharing is not applicable to this article.
